# Exploring the Significance of Vitamin D Levels as a Biomarker in Ear Diseases: A Narrative Review

**DOI:** 10.7759/cureus.54812

**Published:** 2024-02-24

**Authors:** Muhammad Hamayal, Saleh Khurshied, Muhammad A Zahid, Nawal Khurshid, Warda Shahid, Maesum Ali, Hammad Ahmed, Mehrun Nisa

**Affiliations:** 1 Otorhinolaryngology, Pakistan Institute of Medical Sciences, Islamabad, PAK; 2 Ophthalmology, Monash Health, Clayton, AUS; 3 Otorhinolaryngology, Federal Medical College, Islamabad, PAK; 4 Paediatrics, Fauji Foundation Hospital, Rawalpindi, PAK; 5 Medicine and Surgery, Pakistan Institute of Medical Sciences, Islamabad, PAK

**Keywords:** ear nose and throat, vitamin-d deficiency, prognostic biomarker, ear disease, vitamin d level

## Abstract

This narrative review examines the role of vitamin D as a biomarker in ear disorders, including benign paroxysmal positional vertigo (BPPV), otitis media, bell's palsy, Meniere's disease, and hearing loss. PubMed, The Cochrane Library, and Google Scholar were utilized to conduct a comprehensive literature search, and findings were combined from studies from 2014 to 2024. As highlighted in this review, there is a consistent association between vitamin D deficiency and an increased risk and recurrence of disease especially in BPPV and otitis media. Its importance as a prognostic biomarker is emphasized in Bell’s palsy, where higher levels of deficiencies in vitamin D are associated with higher grades of severity on the House Brackmann grading system. Vitamin D deficiency can also lead to sensorineural hearing loss due to its receptors present in the inner ear or its effect on calcium metabolism. Serum levels of vitamin D have also been shown to influence treatment outcome of sensorineural hearing loss. The role of vitamin D in Meniere's disease is unclear as no cause has been identified for the increase in endolymphatic fluid. The findings of this review emphasize the importance of serum vitamin D as a biomarker in ear disorders and advocate for more studies to be conducted to assess the importance of optimal dosing of vitamin D for the progression and outcome of these diseases.

## Introduction and background

Disorders of the ear represent a spectrum of conditions and can be classified depending upon the location of the abnormality into diseases of the external ear extending from the pinna to the outer tympanic membrane, middle ear, i.e., the tympanic cavity, and inner ear involving the cochlea, semicircular canals, and vestibule. These conditions range from acute to chronic and significantly impact hearing, causing conductive or sensorineural hearing loss along with other common clinical features such as otalgia, otorrhea, foul smell, vertigo, nausea, tinnitus, and imbalance depending upon the type and location of the disorder [1,2]. Etiologically, the disorders can be congenital, infectious, traumatic, genetic, and neoplastic. Some common conditions affecting normal ear functioning include otitis externa, congenital external ear disorders, benign paroxysmal positional vertigo (BPPV), Meniere's disease, acute otitis media (AOM), otitis media with effusion (OME), otosclerosis, and bell's palsy as well as a few other disorders [3].

Globally, around 360 million people have ear disorders leading to hearing loss, with children constituting around 32 million of the affected population. According to WHO, these ear diseases cost around 750 billion international dollars worldwide which is quite a burden economically [4]. Of all disorders involving the ear, otitis media (OM) is the most prevalent globally in both high and middle-income countries. Similarly, 60% of the hearing losses are due to preventable causes [5]. Thus, in order to diagnose the disease early and prevent the progression of the disorder to several threatening complications, useful diagnostic and prognostic modalities have been developed and discovered. One of these modalities includes biomarkers. Biomarkers such as otolin-1 have been implicated for their diagnostic use in BPPV, and prestin for early detection of hearing loss [6,7]. Similarly, serum cytokines such as S100A12, IL-10, and ICAM-1 have been thought to be predictive of AOM but further studies are required for confirmation of its use [8]. Considering the use of biomarkers for early detection, prevention of emergence, and prognosis of disease, this review aims to assess the role of vitamin D levels as a biomarker in ear disorders.

Vitamin D, like other nutrients, is essential for normal body functioning. It functions in maintaining bone and muscle health, regulating inflammation, the immune system, and brain development [9]. Owing to its properties and functions, it has been found to be a useful biomarker in several disorders. It identifies high-risk patients for post-stem cell transplantation complications and can act as a biomarker in patients with sepsis. However, the latter is still controversial [10,11]. Vitamin D deficiency has been implicated in increasing the risk and severity of diabetic retinopathy [12]. Similarly, it might also be useful as a biomarker in ulcerative colitis. As a profile marker, vitamin D has found its role in cardiovascular disorders, as its deficiency acts as a risk factor for several cardiovascular disorders [13,14]. Some studies have shown that vitamin D could possibly have a role in ear diseases including Meniere’s disease, BPPV, otosclerosis, and otitis media due to its regulatory effect on modulation of immune response and inflammation as different cells in the body express vitamin D receptors and vitamin D in turn increases antimicrobial peptides as well as expression of genes which help in regulating inflammation and immunity. Even an inverse relation has been associated with vitamin D and inflammation. Similarly, one study reported a decrease in vitamin D levels in patients visiting otorhinolaryngology outpatient clinics [9,15-17]. Thus, considering all the applications of vitamin D as a biomarker, it is essential to see if it has a role in predicting the future of ear diseases as well as in preventing the disease by early diagnosis.

## Review

Methodology

Search Strategy

Databases including PubMed, the Cochrane Library, and Google Scholar were searched for articles with the restriction of dates from 2014 to 2024. The following search terms were used: "Vitamin D", "Ear Diseases," "Benign Paroxysmal Positional Vertigo," "Bell Palsy," and "Biomarkers." Reference lists of articles were also screened.

Selection Criteria, Data Extraction and Synthesis

All types of study designs were included. Studies having full text available, in English as well as having patients suffering from any of the ear disorders were included. However, studies were excluded if they were not in English or had only abstracts available. Studies with patients suffering from parathyroid disorders or any other bone disorders were excluded. Similarly, abstracts from conferences were also excluded. Screening and selection were carried out by three independent reviewers.

A narrative approach was followed. Studies were divided into different groups based on the disorder and their findings related to vitamin D levels and outcome of the disorder were extracted and examined. This was also done by three independent reviewers and any dispute was resolved with the help of a fourth reviewer. We related the levels of vitamin D to the prognosis and outcome of the disease thus providing an extensive narrative review of the current literature.

Review

Overview of Vitamin D

**Figure 1:**
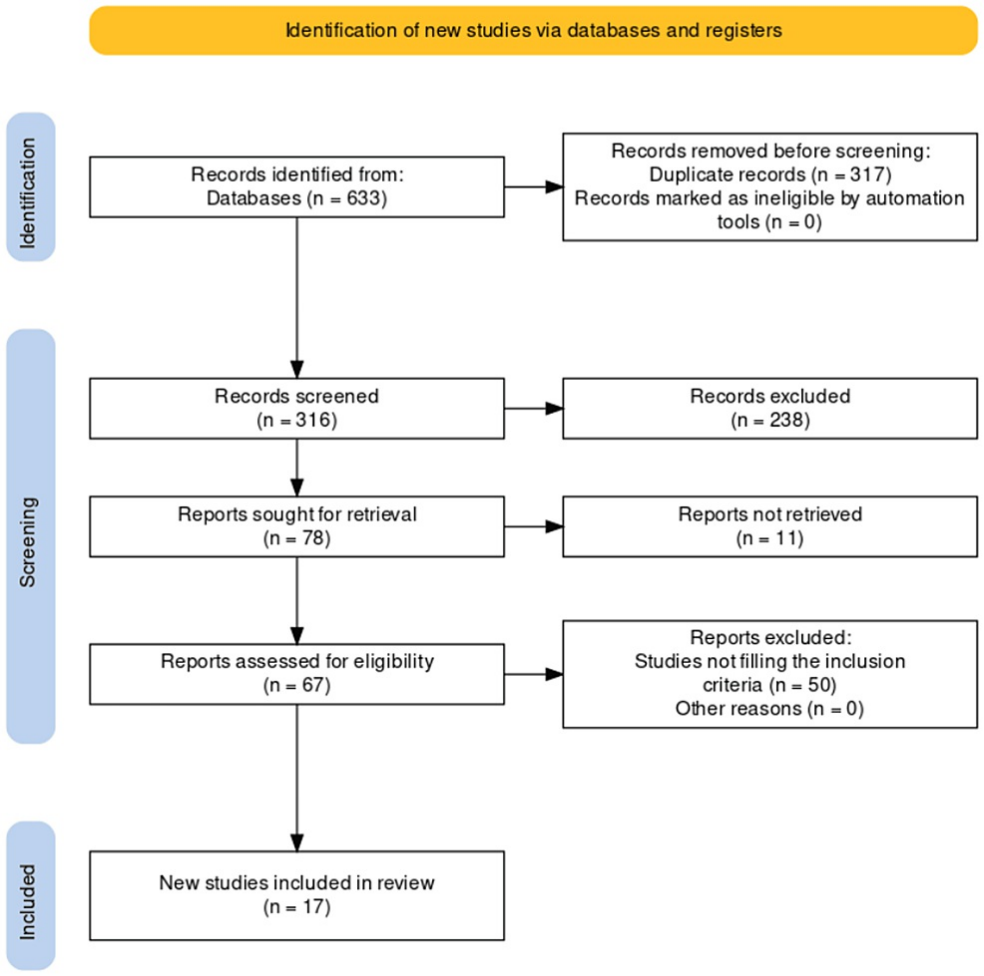
PRISMA flowchart for search and selection procedure of studies PRISMA: Preferred Reporting Items for Systematic Reviews and Meta-Analyses

Vitamin D, a fat-soluble vitamin, is obtained endogenously and exogenously but the natural source is production from skin via sunlight. When UV rays of sunlight fall onto the skin, the 7-dehydrocholesterol is converted into provitamin D3 also known as cholecalciferol which is converted in the liver to 25-hydroxyvitamin D3 (25(OH)D) by hydroxylation. This resultant compound undergoes further hydroxylation in the kidney leading to the formation of 1,25-dihydroxyvitamin D3 (1,25(OH)2D) [18,19]. Although, 1,25(OH)2D is the active metabolite, however, serum level 25(OH)D is considered the best for reflecting the status of vitamin D in the body as it shows a total of vitamin D levels produced endogenously or obtained from exogenous sources. A serum level below 20 ng/ml and below 30 ng/ml for 25(OH)D can be termed as vitamin D deficiency and insufficiency respectively according to the Endocrine Society clinical practice guideline [9,20]. Considering the functions of vitamin D such as strengthening acquired and innate immunity, regulation of inflammation and antimicrobial peptides, and antineoplastic and antiangiogenetic activity, its levels might warrant its role as a biomarker in various diseases [19]. Thus, this review comprises 17 studies to see if there is a role of vitamin D levels as a biomarker in ear disorders. The PRISMA (Preferred Reporting Items for Systematic Reviews and Meta-Analyses) flowchart for study selection is shown in Figure 1.

Vitamin D and BPPV

Benign paroxysmal positional vertigo (BPPV) is one of the leading causes of vertigo and is due to the dislocation of otoconia from the saccule into the semicircular canals, posterior semicircular canal more suitably. A prevalence of 2.6% has been reported for BPPV. As vitamin D plays a role in maintaining the structure of otoconia via calcium levels inside the semicircular canal, a disturbance in vitamin D levels may lead to disruption in calcium metabolism and transportation leading to disruption of otoconial absorption and synthesis [21,22]. Six studies were included which related vitamin D levels to BPPV.

In a case-control study, serum 25(OH)D was measured in 177 BPPV patients taken as cases and compared to controls. The study showed that low levels of vitamin D were associated with a risk of BPPV as shown by a 62% prevalence of vitamin D deficiency in cases as compared to controls. Similarly, a high recurrence rate was found in patients with lower levels of vitamin D. They also reported that females were at a higher risk of BPPV if their vitamin D levels were low [23]. Consistent with these findings, two more case-control studies of 52 cases BPPV cases compared to 52 healthy controls and 69 BPPV cases compared to 68 healthy controls respectively also showed that levels of vitamin D were inversely associated with BPPV with levels being deficient in diagnosed cases. However, no statistically significant differences in 25OHD levels were found between genders and between recurrent and nonrecurrent BPPV [22,24].

In a meta-analysis of 614 patients of BPPV, containing three case-control and four cohort studies. It was interestingly shown that vitamin D levels were not significantly different in BPPV cases compared to control groups. These results although opposite to the current literature might have been affected due to confounders such as respiratory tract infections and osteoporosis as vitamin D levels are lower in such patients. However, despite the presence of confounders, a difference was observed in cases with lower vitamin D levels were prone to recurrence of vertigo attacks [21]. Similar to the study of Jing Ding, this meta-analysis also showed that females with deficient vitamin D levels were at a higher risk of developing BPPV than males [21,23].

In winter people are less exposed to sunlight than in summer, thus low levels of vitamin D can be found in winter making them prone to BPPV as supported by a review of referrals and letters in the UK. It showed that in the months of least vitamin D exposure, the incidence of BPPV increases as compared to the months of high exposure. Thus, vitamin D levels can be strongly associated with BPPV making them predict the prognosis of the disease [25]. However, a study published in 2021 denied any relation between vitamin D levels and the development or prognosis of BPPV. Around 41% of BPPV patients had lower vitamin D levels which were not really different from other disorders. Contradictory to the study of Algarni et al. and Ding et al., this study showed no relation between vitamin D and with recurrence of vertigo attacks in BPPV patients [21,23,26].

Taking the above-mentioned literature into consideration, it can be said that vitamin D has a role in the development of benign paroxysmal positional vertigo and hence can act as a biomarker. Levels of 25(OH)D could be able to detect its prognosis, chances of recurrence, and even the need for supplementation with vitamin D supplements where needed to prevent the BPPV from progressing. Low levels of vitamin D could also be used for screening patients at a higher risk of developing the disease. Summaries of studies on BPPV and vitamin D are in Table 1.

**Table 1:** Summary of studies on vitamin D and BPPV BPPV: Benign paroxysmal positional vertigo

Article	Study design	Study characteristics	Main findings
Algarni et al. [21]	Systematic review and meta-analysis	Sample size = 1015; Consisting of three case-control studies and four cohort studies	No difference in vitamin D levels between BPPV cases and controls; and low vitamin D levels are associated with recurrence
Inan et al. [22]	Retrospective case-control study	Sample size = 104 with 52 BPPV cases and 52 controls	Higher rate of vitamin D deficiencies in BPPV patients
Ding et al. [23]	Cross-sectional study	Sample size = 522 with 174 BPPV cases and 348 normal individuals	Low vitamin D levels in 62.1% of BPPV cases; and low levels of vitamin D in recurrent BPPV
Sarsitthithum et al. [24]	Prospective-cross sectional study	Sample size = 137 with 69 BPPV cases and 68 controls	Low serum 25(OH)D in BPPV patients while no difference in vitamin D levels between recurrent and other patients
Meghji et al. [25]	Retrospective review	Sample size = 339 diagnosed cases of BPPV	BPPV shows seasonal variability with vitamin D being lower in winters
Goldschagg et al. [26]	Short communication	Sample size = 680 with 158 cases of BPPV, 221 cases of vestibular disorders, and 301 cases of non-vestibular disorders	No difference in vitamin D levels between BPPV patients and others

Vitamin D and Otitis Media

Otitis media is the inflammation of the middle ear. It can be classified into three main types. Acute otitis media: Acute suppurative inflammation of the middle ear due to infection secondary to eustachian tube blockade, chronic otitis media: Chronic inflammation of the middle ear lasting for more than three months, and otitis media with effusion: Also known as secretory otitis media (SOM) with fluid in the middle ear. OM in pediatric patients is one of the most prevalent disorders with around 80% of the children below seven years of age getting infected. Similarly, OME has been shown to occur in 90% of children below four years of age [27,28]. Vitamin D has a positive role in strengthening the immune system. It upregulates antimicrobial peptides such as defensins beta-2 and cathelicidin which help the immune system to perform effectively. However, a deficiency of 25(OH)D might lead to impaired immunity and thus an increase in infection [27]. Considering this high-yield role of vitamin D, we were able to include five studies to explain the role of vitamin D as a biomarker in otitis media.

In a prospective controlled study of 174 cases of OME with age below 13 years and 80 children below nine years of age in the control group. This study showed that mean vitamin D levels were below 20 ng/ml in patients of OME as compared to the controls and 39.1% of the cases were vitamin D deficient. Moreover, OME was prolonged in patients with vitamin D deficiencies and had to undergo ventilation tube (VT) placement. This shows that vitamin D has a big role in the prognosis of OME [27]. Similarly, another study design was conducted on children having SOM with adenoid hypertrophy as cases showed significant differences in vitamin D levels being lower in patients of SOM. Gender-wise, females were more likely to have vitamin D deficiency associated with SOM. Furthermore, the adenoids enlarged in patients with vitamin deficiency which could further worsen the prognosis of OME [28].

A meta-analysis of 16,689 individuals showed that vitamin D definitely has a role in the development of acute otitis media and possibly a greater chance of recurrence in 25(OH)D hypovitaminosis. Thus, low levels of vitamin D led to a worse prognosis increasing the chances of recurrent otitis media (rOM). However, no convincing evidence was found to relate vitamin. D levels to chronic otitis media (COM). This study further showed that additional therapy with vitamin D might improve the outcome of the disease. However one should be cautious in dosing so as not to cause hypervitaminosis [29]. The same primary results were shown in another meta-analysis too of 17,614 individuals. However, an interesting finding was observed that patients of OM below five years of age had significantly lower vitamin D levels than patients above five years of age. Patients receiving vitamin D supplementation had lower rates of recurrence as well as lower chances of developing OM, thus preventing as well as improving the outcome of the disease [30].

Research in Egypt containing 30 cases of otitis media also backed the above-mentioned literature showing abnormal vitamin D levels in 22 of 30 cases of OM along with children below six being more likely to have vitamin D deficiency [31]. Therefore, it can be stated that according to the included literature, vitamin D has a very beneficial prognostic and preventative role in AOM and OME. Owing to its microcidal as well as immune system enhancement effects, the role as a biomarker is imminent. Especially every child with OM should be tested for serum 25(OH)D levels so as to not worsen the prognosis due to vitamin deficiency. The included articles on OM are summarized below in Table 2.

**Table 2:** Summary of studies on vitamin D and otitis media AOM: Acute otitis media; COM: Chronic otitis media; OM: Otitis media; OME: Otitis media with effusion; rAOM: Recurrent acute otitis media; RCT: Randomized control trial; SOM: Secretory otitis media

Article	Study design	Study characteristics	Main findings
Akcan et al. [27]	Prospective controlled study	Sample size = 254 containing 174 OME cases and 80 controls.	Vitamin D deficiency in OME patients with more chances of VT in vitamin D deficient individuals
Mandour et al. [28]	Prospective case-control study	Sample size = 150 with 100 SOM cases and 50 normal individuals	SOM was more common in winter; low levels of vitamin D were found in cases associated with enlarged adenoids
Li et al. [29]	Systematic review and meta-analysis	Sample size = 16689 constituting two case-control studies, one RCT, one observational, and one prospective study	Low vitamin D levels in AOM; vitamin D hypovitaminosis in rAOM; additionally, treatment of OM with vitamin D is possible
Salamah et al. [30]	Systematic review and meta-analysis	Sample size = 17614 with one RCT, one prospective, one observational, and seven case-control studies	AOM is associated with vitamin D deficiency while COM has a weak association with vitamin D; similarly, children below five years with OM had lower vitamin D compared to rest
Salem et al. [31]	Prospective case-control study	Sample size = 40 containing 30 AOM cases and 10 controls	Abnormal vitamin D levels in AOM patients with vitamin D deficiency more common in children below six years

Vitamin D and Bell’s Palsy

Most of the cases of facial paralysis constitute Bell's palsy which is idiopathic unilateral facial nerve palsy. Other causes such as infection, congenital, and trauma are known but Bell's palsy is by far the most common. Although the disease is self-limiting around 30% of the cases may get worse progressively leading to poor long-term outcomes such as facial weakness, deformity of face, and pain [32]. Clinical features such as hyperacusic chewing and speech as well as problems of tearing, taste, and salivation can be explained using the mixed nature of facial nerve [33]. It is established that vitamin D induces nerve recovery and regeneration. It is also involved in the myelination of nerves via the regulation of genes such as Prx and Tspan2. Neurotrophins have a role in maintaining the function of neurons as well as helping them survive and vitamin D has a role in the stimulation of neurotrophins [34-36]. 

In a study containing 52 cases of Bell's palsy, it was reported that vitamin D levels were lower in the patients compared to the control group. Similarly, according to the House Brackmann grading system, higher grades having more severity had low levels of vitamin D compared to patients in the early stages of the disease. So, vitamin D deficiency might have a role in the development of Bell’s palsy and worsening its prognosis as it stimulates neurotrophins involved in sustaining nerve function, and regulates genes involved in myelination and nerve regeneration. Thus, vitamin D deficiency might disrupt these functions leading to Bell’s palsy [32,35]. In another study, similar results were reported where patients with Bell palsy had lower serum 25(OH)D and higher vitamin D deficiencies leading to an increase in the severity of the disease. Moreover, recovery rates were poor in patients having less than 10 ng/ml of vitamin D [35]. Along with facial palsy, hemifacial spasm which is also a dysfunction of the facial nerve characterized by hyperactivity showed a decrease in vitamin D levels according to one study we found and might be due to the demyelination effect of vitamin D [37]. Summaries of these studies are shown in Table 3.

**Table 3:** Summaries of studies on vitamin D and Bell's palsy and hemifacial spasm BP: Bell's palsy; HFS: Hemifacial spasm

Article	Study design	Study characteristics	Main findings
Uysal et al. [32]	Retrospective case-control study	Sample size = 102 containing 52 BP cases and 50 controls	Vitamin D deficiency in BP patients was lower compared to controls. Similarly, high-grade BP patients had lower vitamin D levels compared to low-grade BP patients
Ocak et al. [35]	Prospective case-control study	Sample size = 91 with 43 BP cases and 48 normal individuals	No significant difference in vitamin D between both groups; however, poor outcome was observed in vitamin D-deficient individuals.
Ulusoy [37]	Prospective case-control study	Sample size = 160 with 80 BP cases and 80 normal individuals	Low vitamin D levels in HFS compared to controls. But no effect of vitamin D on the severity of the disease

Considering these studies, vitamin D levels need to be measured as early as possible to avoid worsening of facial palsy as higher deficiencies are associated with higher grades of severity. Similarly, supplementation where required could help the patient recover early. Thus, as a prognostic biomarker, vitamin D could play a very essential role and improve the overall outcomes.

Vitamin D and Meniere's Disease

Meniere's disease is still a matter of debate as no cause has been confirmed for the increase in endolymphatic fluid. Many possible etiologies have been suggested. Some are autoimmune, genetic, and even due to allergies [38]. The only study included in this review showed vitamin D levels were associated with endolymphatic hydrops [39]. But this association could be more due to the effect of vitamin D in causing hearing loss, tinnitus, and vertigo as discussed in the section on hearing loss below which are the clinical presentations of Meniere's disease rather than the disease itself. However, if the autoimmune etiology of Meniere’s disease is considered, a deficiency in vitamin D could lead to its development [9]. A summary of the included study is shown in Table 4.

**Table 4:** Summary of study on vitamin D and Meniere's disease MD: Meniere's disease; OR: Odds ratio

Article	Study design	Study characteristics	Main findings
Bakhshaee et al. [39]	Case-control study	Sample size = 112 containing 28 MD cases and 84 controls	Deficiency of vitamin D in MD cases compared to controls. Additionally, vitamin D was inversely related to MD according to OR

Further studies are required to assess vitamin D supplementation to improve outcomes of Meniere’s disease.

Vitamin D and Hearing Loss

Hearing loss is a common condition with around 15% population affected globally being more prevalent in the elderly. It can be conductive or sensorineural depending upon the cause. Vitamin D deficiency can lead to sensorineural hearing loss due to its functions in the inner ear due to its receptors or role in calcium metabolism [40]. Three studies assessing the role of vitamin D have been included in this review.

In one study by Szeto et al., low vitamin D levels were associated with low and speech-frequency hearing loss [41]. As low vitamin D levels can alter calcium levels via disruption of metabolism and absorption thus hindering normal hearing as calcium is needed for the conduction of electrical impulses. However, calcium levels were found to be normal as compared to serum 25(OH)D levels. No significant association was found between high-frequency hearing loss and levels of vitamin D. A different finding from all other studies included yet was the response of disease to treatment. Surprisingly an increase in vitamin D levels was related to a better response towards steroid therapy for hearing loss which adds to the role of vitamin D as a prognostic biomarker in hearing loss as well as other ear diseases to improve the outcome of disease [41].

Similarly in another study by Ghazavi et al., sudden sensorineural hearing loss was assessed in relation to vitamin D. Significantly, the same trend as has been followed in this review was reported. Vitamin D deficiency was strongly affiliated with hearing loss. The needed findings for vitamin D to be used as a biomarker were that in recovered patients’ serum 25(OH)D levels were higher as compared to patients who didn’t recover. Although the effect of supplementation was not shown, treatment response was better in comparison to patients with low vitamin D levels. Thus, fulfilling the criteria to be effective as a biomarker as its low levels make the person prone to hearing loss and its increasing levels propose a better outcome [40]. Summaries of both studies are shown below in Table 5.

**Table 5:** Summary of studies on vitamin D and hearing loss NHANES: National Health and Nutrition Examination Survey; SSNHL: Sudden sensorineural hearing loss

Article	Study design	Study characteristics	Main findings
Ghaznavi et al. [40]	Cross-sectional study	Sample size = 68 with 34 SSNHL cases and 34 normal individuals	73.5% of patients of SSNHL had low vitamin D levels and better treatment response was observed in patients with high vitamin D levels. Similarly, patients with sufficient vitamin D showed complete recovery compared to the rest
Szeto et al. [41]	Review of cross-sectional surveys obtained from NHANES	Sample size = 1123 with mean age = 76.4 ± 0.13	Age-related low-frequency and speech-frequency hearing loss are associated with vitamin D deficiency

## Conclusions

Our review has extensively shown the role of vitamin D in several ear diseases. Vitamin D has the ability to act as a powerful prognostic biomarker as well as to prevent the development of the disease in high-risk individuals in diseases such as benign paroxysmal positional vertigo, otitis media, Bell’s palsy, and hearing loss. Levels of serum 25(OH)D have been able to predict the outcome of disease with lower levels leading to worse progression. Therefore, patients of these four diseases should almost always be assessed for vitamin D levels so as to enhance the positive outcomes via timely management especially in Bell’s palsy and otitis media with a tendency to get complicated. Its supplementation has also been shown to improve recovery of the patients; however, in addition to longitudinal studies with extended follow-ups to evaluate therapeutic implications, more trials are required to determine the ideal dosage, effectiveness, and safety of vitamin D supplementation in ear disorders. Additionally, vitamin D’s role in Meniere’s disease wasn’t convincing due to its unknown etiology so further studies are needed in this area too.
